# Qualitative Evaluation of the Implementation of an Integrated Care Delivery Model for Chronic Patients with Multi-Morbidity in the Basque Country

**DOI:** 10.5334/ijic.1975

**Published:** 2016-08-19

**Authors:** María Luz Jauregui, Carmen Silvestre, Pedro Valdes, Idoia Gaminde

**Affiliations:** 1 Clinical Management Unit, Tolosaldea Integrated Health Organisation, Basque Health Service (Osakidetza), Spain; 2 Quality and Clinical Safety Unit, Servicio Navarro de Salud-Osasunbidea, Spain; 3 General Practitioner, Deusto Primary Health Centre, Osakidetza, Basque Country, Spain; 4 Research, Innovation and Knowledge Management Unit, Department of Health, Government of Navarre, Spain

**Keywords:** integrated care, family medicine, multi-morbidity, qualitative evaluation

## Abstract

**Introduction:**

The objective of this study was to assess a model for improving healthcare integration for patients with multiple chronic diseases in an integrated healthcare organisation in the Basque Country and to propose areas for improvement.

**Methods:**

We organised four nominal groups composed of representatives from different categories of clinicians involved in the development of an integrated healthcare organisation and in the integrated care of patients with multiple diseases, namely, internists, general practitioners, and primary care and hospital nurses.

**Results:**

The aspect rated most positively was the concept itself of an integrated care model, which is able to improve communication between levels of care, increase the quality of the care provided and enhance patient safety. Additionally, it was agreed that the role of assigned clinicians is a key element. The problems identified mostly concern its implementation in daily practice.

**Conclusions:**

The results of this study made it possible to suggest at least 8 areas of improvement to be implemented. These are related to: nurses’ roles; care and monitoring of stable patients; team work; communication with patients; coordination with social workers and between internists and family doctors; as well as the development of an office of medical services to lead the integration process.

## Introduction

One of the implications of ageing, from a clinical point of view, is an increase in the number of people with two or more chronic diseases. Life expectancy for the Basque population has extended considerably in recent decades and a significant parallel change has taken place in life styles. One consequence of this is that the prevalence of people suffering from chronic illnesses is increasing to the extent that the great majority of patients in our health system are suffering from one or more chronic illnesses. Hospital records clearly show not only that the rate of hospital attendance is higher in older people, but also that their mean hospital stay tends to be longer than that of younger patients. In addition, they use more resources in terms of medical consultations, emergency room attendances, day hospitalisation and medications [1]. Moreover, about 80% of interactions with the health care system and 77% of health expenditure in the Basque Country are due to chronic conditions [2].

Elderly patients represent a high proportion of the care load and are high users of healthcare resources, with associated cost implications [3,4]. In the Autonomous Community of the Basque Country (Spain), only 23% of those older than 65 years report being free of chronic conditions, whereas 37.5% declare having 2 or more conditions [5].

This situation poses a problem: if we provide care to patients with chronic diseases and those with social and/or long-term, low-intensity healthcare needs in hospitals with resources for acute illnesses, we may not be able to maintain the balance between access and quality, on the one hand, and the sustainability of the healthcare and social systems, on the other. The current organisational model – mainly focused on acute hospital care – must be able to adapt, as well as to adopt tools to face the aforementioned types of social changes and demands [1,6]. The current organisational model is not ideal for delivering care to these patients; indeed, it fails to meet their needs, which include continuity of care, prevention of dependence, and stopping or slowing the progression of the diseases they already have.

To improve the quality of care provided to patients with multiple chronic health problems, as well as efficiency in the management of health and social care resources available, there is a need to manage their care in a systematic manner, based on integration and coordination across all the different levels of care. Given all this and following the strategy of the Department of Health of the Government of the Basque Country, it was suggested that new organisational healthcare models were required, redefining care pathways to improve coordination and avoid inefficiencies in the health service [3].

This new strategic approach is supported by international trends and by the increasing evidence of the effectiveness of the interventions and models more in tune with the needs of chronic patients. The Basque strategy is presented as an opportunity for change in the acute care model to one in which the agents involved participate in the establishment of a framework of action for an integral management of chronicity from the population perspective, building upon the existing capabilities of the system [2].

The change management agenda was focussed on introducing new models of care which helped to create ‘local systems of care’. The models of care that were used were based on the Chronic Care Model [7], the Triple Aim [8,9]; and risk stratification. These frameworks and models are well known, but the important issue is to signal their relevance in a complex change process since they stimulate ‘system’ thinking and open the door to a population health perspective. Using these frameworks it becomes possible to target interventions and preferably address those more vulnerable patients at risk of future hospitalisations [3].

Presently the whole population of the Basque Country is stratified according to its risk of hospitalization; in the microsystems where it is being used this is a key factor in the redefinition of the work among team members [3]. At the delivery level, the Basque health care system did not have the essential elements required for coordinated or integrated care, and it was clear that few delivery organisations in the Basque Country were using any of the possible care management processes which support integration of care. Therefore care management processes (i.e. ‘integrators’) were developed to ensure that all had the potential to reinforce integration of care.

The goal was the coordination and integration of organisations at a local level in clinical terms rather than a focus on structural or managerial integration. Furthermore the intention was to seek alignment of both top-down and bottom-up ‘integrators’, an alignment based on their integrative potential. Bottom-up interventions sought to engage clinical and nursing leadership in the change process. The search for new approaches of clinical leadership requires a much greater involvement by health care professionals in the overall effectiveness and performance of the health care system.

Top-down interventions followed a more traditional and formal planning approach in view of the fact that they needed to be standardised across the entire health care system. For example, like many other health care systems the Basque resource allocation system was actually financing fragmentation. That is to say, resources were allocated to the providers of the system individually – hospitals, primary care centres, social services – to carry out activity. A new approach to joint commissioning (bundled payments across primary and hospital care) was launched to encourage coordinated work at the provider level and to incentivise innovation in local care delivery.

The Goierri-Alto Urola Integrated Healthcare Organisation (IHO) is one of such new integrated organisations in the Gipuzkoa Health Region, part of the Basque Health Service (Osakidetza). It is composed of a regional hospital and 7 primary care centres, with a catchment population of over 98,000 people. It should be highlighted that the two levels of care brought together under this organisation, primary care and the hospital, had different management teams with different focuses, and that the culture of professionals is very different in the two settings [10]. This IHO took part in a pilot study to test for different ways of delivering care for patients with multi-morbidity, those at the top of the risk stratification. Specifically, the aim was to integrate care for patients with multiple diseases and/or on multiple medications, by introducing the role of assigned internist and consultant, and piloting two new nursing roles requiring new nursing competencies: a) liaison nurses based in the hospital, and b) advanced practice case managers based in ambulatory care whose role was to coordinate GPs, primary care and liaison nurses on behalf of chronic patients [2].

The objective of the study is to evaluate from the participants’ point of view the model of integration for improving healthcare integration and care for patients with multiple chronic diseases in the Goierri-Alto Urola Integrated Healthcare Organisation and propose areas of improvement.

## Methods

Using the nominal group technique [11], four groups of professionals were run with representatives of different categories of clinicians involved in the development of the integrated healthcare organisation and in the integrated care of patients with multiple chronic conditions: internists, general practitioners, and primary care and hospital nurses (including those delivering the hospital-at-home services). The research team produced a document to generate discussion between the clinicians invited to participate, this being circulated in advance of the group sessions. It contained a summary report of the pilot phase and set out the key questions to be addressed, in order that participants could work on them before addressing them in the groups. In all the group sessions, the following questions were considered: 1) ‘What is the best/most positive aspect of the model implemented?’ 2) ‘What is the worst/most negative aspect of the model implemented?’ and 3) ‘How could the model be improved?’ seeking proposals for improvement to guide the development of a healthcare integration model for patients with multiple chronic diseases. First, the participants had to generate ideas – silently without discussion – and write them on index cards; one card is used per idea. Second, the facilitator collected the cards, registered every idea on a flip chart, so that team members could briefly discuss the ideas for clarification and eliminate similar ideas from the chart. Third, ideas were discussed to determine clarity and importance. For each idea, the moderator asked, “Are there any questions or comments group members would like to make about the item?” This step provided an opportunity for members to express their understanding of the logic and the relative importance of the item. It made it possible to group ideas as well. The creator of the idea did not feel obliged to clarify or explain the item. Finally, participants rated the ideas (on a scale ranging from 0 = not important at all to 5 = very important).

The sessions were held on 29 and 30 May 2013 in the boardroom of Zumarraga hospital, and lasted 3 hours -around one hour per question posed-. The clinicians and nurses invited to participate in each group were selected to ensure that they were key informants.

In the analysis, we considered the content of the proposals and other ideas put forward in each of the groups. Specifically, we considered the strength of the ideas in terms of the number of times they were repeated individually and spontaneously (spontaneity) and of their ranking by participants, an indirect measure of their relative importance, together with the variability in the degree of agreement between the participants in these rankings (a coefficient of variation, with higher values reflecting greater heterogeneity in the ranking by group members, and lower values more homogeneity).

## Results

Seven general practitioners, 7 hospital nurses, 8 internists and 10 primary care nurses participating took part in each group. All GPs were involved in the original study on the new roles, half of them in the test group, and the other half in the standard care one. The hospital nurses’ group was composed of 2 nurses performing the new roles (liaison nurse and advanced practice case manager), 1 internal medicine supervisor, 2 home care nurses and 2 internal medicine nurses. All internists had been “internists of reference” in the pilot study. And, finally, primary care nurses took part in the pilot study and had to collaborate with liaison nurses; 3 of them were mangers at their teams.

### Primary care nurses

In response to the first question, concerning positive aspects, participants put forward 55 ideas (mainly focused on theoretical virtues), which after clarification and the sharing of views yielded 13 themes. In three cases, there was complete consensus on their importance: the model improves the quality of care, enables a better assessment of patients, and facilitates management of their conditions; this last point means an increase in patient participation in the management of their own health, and the production of joint management plans, between levels of care, between different categories of professionals and with social services. These themes are directly related to better communication and the integrated nature of the patient-centred model: “We all know each other”, “Now, we are all more aware of the complexity of the situation” and “The patient belongs to all of us”. The role of assigned clinicians, namely, advanced practice nurse case managers, hospital liaison nurses and internists, was also considered positive.

For question 2, asking about negative aspects, the 36 ideas generated were grouped into 9 themes. There was consensus on the low level of commitment of assigned internists, this generating eight different ideas on which there was complete consensus; and on weaknesses of the information technology support systems, possibly linked to the lack of a shared electronic record. Despite the pilot having opened new channels of communication, participants identified problems of coordination between primary and specialised care, such as incompatible working hours in the different places of work, poor communication, waiting times for patients in the emergency department, and the time of day when patients are discharged. Problems were also detected regarding resistance to change by some professionals and a lack of protocols for stable patients with multiple chronic diseases; these are attributed respectively to poor planning of the initial training, and a lack of clear guidelines for once patients have stabilised and their care is transferred to the primary care provider. This analysis also revealed problems that have arisen since the pilot study was completed, specifically related to two themes: the perception of patients regarding continuity- they had not realised that the pilot was a time-limited study- and changes in the services provided by professionals during the study. Finally, we should not forget that there was also resistance, hindering change: “Lots of changes in little time”.

Regarding proposals for improvement (Table 1), 33 ideas were put forward and these were grouped into 11 themes. There was complete consensus on the need for a shared electronic record; as well as on the need to all work together in a team, in all senses, including community and social services. The third one being the need to improve the communication amongst all stakeholders, and above all, with patients; and to develop protocols to ensure continuity of care, and hence the need for all to be committed and responsible. In relation to this, there was a call to reinstate key elements of the pilot study, namely, the assigned clinicians, providing stability, and continuity of the model. Additionally, a new figure was proposed: “a project champion, to manage conflicts and resistance in the organisation”.

**Table 1:** Primary care nurses. Proposals for improvement.

Themes	Ranking	Coefficient of variation	Spontaneity

Shared health record	5.00	0.0	5
Team work between specialised care, primary care and social services (protocols, care, discussion, etc)	5.00	0.0	7
Motivation, commitment, and the taking on of responsibility among professionals	5.00	0.0	4
More resources	4.67	0.11	4
Efforts to improve communication with patients	4.22	0.16	4
More information for staff about the model and continuity of the model	4.22	0.2	2
Remote consultations between clinicians	4.22	0.2	1
Assigned clinicians	3.89	0.24	3
Project champion, to manage conflicts and resistance in the integrated health organization	3.63	0.31	1
Data on clinical outcomes by doctor’s list	3.56	0.25	1
Videoconferences	3.11	0.41	1

### Hospital nurses

A total of 20 ideas were generated regarding the most positive aspects of the healthcare integration model. On this question, there was no consensus on the importance of several themes, but the figures of advanced practice nurse case manager and liaison nurse were the most highly valued, affirming the importance of these new nursing roles. Other positive aspects identified include the promotion of self-management among patients; the importance of taking a holistic view of patients, as well as patient satisfaction, and the possibility of having access to a shared electronic health record. They also referred to benefits such as the ability to avoid unnecessary transfers and to plan admissions; focusing, above all, on the potential power of the model compared to the status quo, after completion of the pilot study.

Concerning the most negative aspects of the model, 27 ideas emerged, and these were grouped into 13 themes. As for primary care nurses, these focused what had been lost after the pilot, namely, changes regarding the figure of the advanced practice nurse who was no longer health-centre based, but rather in the hospital-at-home service. Indeed, in the proposals, the status quo is indicated to illustrate the differences.

They referred to a lack of time (now), difficulties with communication between levels of care; and problems that have arisen from a lack of commitment among colleagues. Now, “patients are less well monitored”.

Regarding proposals for improvement (Table 2), the 20 ideas put forward were grouped into 9 themes. These focused on reinstating the figures of the advanced practice nurse case manager and liaison nurse, and the role other professionals, and levels of care, need to play for the model to be successful, above all the role of internists. Another proposal was to extend the healthcare integration model to other patients beyond those with multiple health problems.

**Table 2:** Hospital nurses. Proposals for improvement.

Themes	Ranking	Coefficient of variation	Spontaneity

Internists to take on their role as case managers	4.86	0.08	1
Unification of criteria for action (establishment of protocols for actions, etc.)	4.86	0.08	4
Greater commitment from all levels of care	4.86	0.08	3
Reinstatement of the figures of the advanced practice nurse case manager and liaison nurse in primary care, or taking on of corresponding functions by a primary care nurse	4.86	0.08	3
More staff, to avoid work overload	4.57	0.17	3
Differentiation of the tasks of each professional	4.57	0.12	2
Information technology tools to simplify record-keeping	4.00	0.14	2
Simpler model, based on protocols, that cover more patients (beyond those with multiple health problems)	3.86	0.31	1
Grouping patients with multiple chronic health problems on specific doctor’s lists	3.29	0.29	1

### General practitioners

Unlike the other three groups, the general practitioners decided to consider the underlying concepts, more than their personal experience. The total of 19 conceptual ideas generated were grouped into just 4 themes providing a very positive assessment of the proposal of integrated healthcare for this type of patients, and identified a series of advantages, including an improvement in the relationship between levels of care, and the usefulness of the figure of the assigned professional for this type of patient.

In the second question, they focused entirely on their experience in the integrated healthcare organisation pilot study. They suggested 46 different ideas, yielding 15 themes. The figure of the assigned internist was the least valued aspect of the pilot study, as it was felt that their colleagues did not fulfil the role they were given, and this was associated with a lack of coordination and collaboration, and possibly also with their statement that the model was not successful despite being “an ideal to pursue”.

With regards to the role of the internist, they sensed little commitment, a lack of coordination, and poor communication, among other problems. This was directly linked to the lack of shared goals, and a feeling that the tasks assigned to general practitioners were poorly defined. They also described the role of primary care nurses as missing from the process, their activities having been insufficiently visible.

They indicated a further series of problems during the implementation: weak problem-solving capacity; a lack of consensus on prescribing; the questionable sustainability of certain figures in the new model (in particular, the advanced practice nurse case manager and liaison nurse); and a lack of overlap in working hours between all those involved, etc. They identified certain key elements in a healthcare integration policy that had not been addressed in the first phase, namely, long-term, low-intensity social and health care, which is a key requirement for this type of patient, and hospitalisation at home. They recognised a need for management of potential disagreements between different categories of professionals. Finally, they raised the issue of the concurrent implementation of different projects as part of the strategy for tackling chronic diseases in the Basque Country.

For the development of an improved integrated care model (Table 3), 57 ideas were generated and grouped into 16 themes. Most were conceptual proposals concerning what an integrated healthcare model should include to ensure that the care provided to all patients, not only those with multiple health problems, is fully integrated. They proposed that clear protocols should be developed and care standardised with shared goals (for all levels of care and categories of clinicians) related to agreed criteria for patient care, and who decides and how; an internal coordination plan should be drawn up for primary, nursing and social care; and the functions and tasks of all the stakeholders should be defined, and provided with the necessary support, such as joint training, and a commitment on the part of the internist. They mentioned the need for the care to be centred on patients and their families, placing the patient at the heart of the care provided. Finally, they pleaded for communication, communication and communication.

**Table 3:** General practitioners. Proposals for improvement.

Themes	Ranking	Coefficient of variation	Spontaneity

Development of protocols and standardisation of healthcare with shared goals, deprescribing	5.00	0.00	15
Commitment of the internist: he/she should be a clinical leader and really want the role	4.86	0.08	5
Joint training – criteria for stability/instability Communication, communication and communication	4.71	0.16	4
Establishment of flexible mechanisms to facilitate communication across the organisation (setting aside time, regular meetings between levels of care, joint meetings)	4.71	0.1	7
Definition of tasks and roles	4.57	0.12	9
Strengthening the role of patients and their families within the programme for patients with multiple chronic diseases	4.14	0.09	4
Real development of information technology tools and electronic health records with automatic reminders and prompts	4.14	0.17	2
Development of the skills of primary care nurses for monitoring patients with multiple chronic diseases	4.14	0.17	2
Coordination with hospital-at-home services	4.14	0.26	2
Encouragement of a greater involvement of general practitioners in the project	4.14	0.17	1
Integration with social services	4.00	0.14	1
Minimisation of unnecessary hospitalisation at home	3.86	0.23	1
Up-to-date records of patients with multiple chronic diseases in the integrated health organisation, discharge criteria	3.71	0.20	1
Liaison nurse pursuing the goals of specialised and primary care	3.71	0.20	1
Management prioritisation of programs, so that they can be addressed properly	3.57	0.15	1
Access by primary care staff to records of hospital follow-up of patients with multiple chronic diseases	3.14	0.29	1

Regarding the establishment of flexible mechanisms to facilitate communication they indicated a need to improve access to the hospital, genuinely bringing the hospital and primary care closer; as well as a need for rapid and real-time communication (e-mail, telephone, etc.); joint sessions between internal medicine and primary care, to discuss and reach a consensus on patients with multiple chronic diseases; regular meetings between different levels of care; and the assignment of a general practitioner to coordinate meetings (with the internists).

### Internal medicine specialists

In the internist group, 28 proposals were suggested, and these were grouped into 8 themes. The most important one was the relationship in terms of communication and/or coordination with primary care: getting to know one another, and the establishment of a cycle of communication (telephone calls, joint clinical sessions). Other positive aspects suggested were a change in mentality of clinicians towards patients with multiple chronic diseases and the figure of the assigned internist, this being professionally very important. Further, they mentioned the optimisation of resource use and colleagues’ ability to adapt to new roles and functions.

With regards to the most negative aspects, the 25 different ideas generated were grouped into 9 themes. Barriers identified mostly concerned a lack of real support and commitment from some health professionals, the great differences between integrated health organisations in Osakidetza, and internal problems within the department, not all colleagues having responded in the same way. It is important to point out that they recognised a lack of leadership on their part and that they, as a professional group, had not been sufficiently involved in the project. Another of the themes related to the feeling they had had a project imposed on them that was poorly explained, with poor leadership and no opportunity for clinicians to express their opinions.

A total of 21 ideas emerged as proposals for improvement (Table 4) and these were grouped into 13 themes. The top one was directly related to one of the identified problems, and concerned the need to provide more resources and tailor them to the requirements of the new model. They requested the involvement of clinicians at an earlier stage, that is, they felt they should be consulted in the planning of new proposals for care, underlining that such proposals need to be adapted to the real state of the care system. They also suggested that care for patients with multiple chronic diseases should be distinguished, as a specific model of care, from the routine healthcare tasks within their department. Finally, they saw a need to optimise the interaction between levels of care, focusing on joint clinical sessions, which would improve communication.

**Table 4:** Internal medicine specialists. Proposals for improvement.

Themes	Ranking	Coefficient of variation	Spontaneity

More resources tailored to needs	4.25	0.24	5
Listening to clinicians from the organisation at the planning stage	4.00	0.27	2
Definition of priorities [defining roles in a realistic way, distinguishing care of patients with multiple chronic diseases from routine practice)	3.75	0.37	4
Official recognition within the organisation of the model and the professionals involved	3.75	0.31	1
Implementation of equivalent models in all the integrated healthcare organisations	3.75	0.24	1
Strengthening the interaction between levels of care: joint sessions, communication	3.25	0.14	1
Sharing of data on the results of the project	3.25	0.39	1
Being realistic about the expected results	3.00	0.36	1
Greater commitment by “some” general practitioners	2.88	0.29	1
Inclusion of internists on the hospital-at-home team	2.75	0.32	1
Improvement in the provision of care in the emergency department and its relationship with the hospital-at-home service	2.63	0.35	1
Encouragement of remote consultations between clinicians	2.50	0.37	1
Prioritisation of programs: “everybody complies with a clear business plan”	2.00	0.40	1

## Discussion

In the opinion of all the groups consulted, the most positive aspect of the new policy is the proposal itself of a model for integrated healthcare. Among the general practitioners, there was agreement that this was an ideal to pursue, while they were highly critical of the pilot study itself. That is, they approved of the idea of providing integrated healthcare to patients with multiple chronic health problems, but underlined that it matters how, an issue we will discuss below together with the proposals for improvement. Overall, it seems that the integrated model studied is capable of improving communication between different levels of care, increasing the quality of the care provided and enhancing patient safety. The figure of the assigned clinician is also perceived to be a key element.

Regarding the most negative points, there were marked differences between groups, based on their experience of the pilot study. Primary care nurses did not have a leading role in the piloting. In contrast, among the hospital nurses, there were individuals who had also been assigned clinicians during the pilot and, at the time of this research, had two different roles in the hospital, as haemodialysis nurses and as case managers for patients with multiple chronic diseases; this led to dissatisfaction among the nurses and confusion among patients. As noted earlier, the general practitioners opted to brainstorm the positive aspect of the integrated care model as an ideal, but the negative aspects they identified were based on their experience during the pilot. Unlike in other groups, among the internists there were representatives from different integrated healthcare organisations, and this is produced subtle differences in their assessments potentially linked to different experiences. Given these differences, it is important to note which group made each of the key negative comments.

Overall, the results reflect the frustration of primary care doctors and nurses with a lack of coordination, communication and commitment. They unanimously criticise internists, who we should recall had a key role in the piloting, for their low level of commitment. This may explain other problems mentioned, such as the lack of coordination.

In practice, the task of coordination between levels of care is far from being simple and orderly, but rather is associated with a certain level of complexity and potential confusion, given the nature of the care activity itself, with great differences between professional groups and their sequential and mutual interdependence, compounded by uncertainties in the clinical and social context [12,13]. The strategies in the literature for improving coordination show the great importance of facilitating mechanisms based on mutual adaptation [14,15], suited to the coordination of healthcare, and exchanges between the professionals involved to solve problems where the relevant information is generated (integrated information systems, informal communication, telephone contact, working groups, joint sessions and liaison devices). In general, mechanisms based on information and communication technologies (electronic health records) and the standardisation of processes and skills (shared protocols, patient pathways, referral guidelines, and expert systems) [14,15] are also perceived as facilitating factors, above all in that they favour communication and consensus between professionals [16,17]. Under this scenario, communication, mutual knowledge and good relationships between clinicians at different levels of care represent the core determinants of good coordination [17]. In contrast, weak leadership and commitment, the lack of shared goals, poor planning, inadequate incentives (usually financial), and overly fragmented organisational and cultural structures are barriers to healthcare coordination [15,18].

Participants from primary care (nurses and general practitioners) raised an issue that merits discussion, namely, the lack of protocols for cases referred to during the pilot as a “stable patients”. That is, what should be done once the hospital care team discharges a patient who has stabilised: How often and how should they be monitored? What guidelines should be followed to keep them stable?

To address chronicity, most health systems propose care pathways based on clinical protocols focused on the main chronic diseases. In order to simplify the approach, linear models are often developed that divide healthcare into smaller units, with the goal of specifying accurately which intervention should be performed for each clinical condition, in line with the mental model that considers the human body as a machine and illness as a malfunctioning of its parts [19]. However, when dealing with patients with more than one health problem, the most difficult but also a very common situation is multimorbidity; to manage such complicated clinical situations, it is usually necessary to establish a diagnostic and therapeutic plan based on a complex multidimensional assessment [20]. For example, in the event of worsening, there tends to be deterioration in the function of various systems, sometimes with vague signs and symptoms. Additionally, we should underline that in primary care a considerable part of daily practice is associated with poorly defined symptoms and as-yet-undiagnosed diseases, which is distressing for patients and stressful for clinicians [21].

It would seem logical to suppose that, as suggested by primary care nurses and general practitioners in this study, tackling these complicated situations requires the design of complex interventions, such as the establishment of healthcare protocols focused on specific subgroups, such as patients with illnesses associated with certain comorbidities. Decision support aid for the guideline-based management of patients with multimorbidity is a challenge since it relies on the combination of single-disease clinical practice guidelines (CPGs). In practice, it is unrealistic and impractical to propose the development of additional clinical protocols covering all the potential combinations of clinical and social circumstances of patients [22]. In patients with multimorbidity, there is usually a high degree of diagnostic uncertainty, and the complexity is compounded by the challenge of decision-making in a context for which it is difficult to develop protocols [12]. Applying clinical guidelines for each of the diseases diagnosed is not a good solution. Attempting to follow all recommendations in all the guidelines would imply performing a vast number of clinical tasks, generating such an absurd amount of work and complexity that both patients and clinicians would be likely to fail to adhere to the plan. As Roland concludes: “multimorbidity introduces clinical uncertainty in a way that is unlikely to be resolved by ever more sophisticated guidelines” [23].

However, there are conceptual models that attempt to capture and define the basic characteristics of complexity in the care for patients with multimorbidity. In general, they address the presence of multiple factors with an impact on patient health, from the comorbid clinical conditions themselves to social and long-term care needs, and the healthcare organisation, highlighting the importance of interaction between patients and providers as a critical and dynamic influential factor [24,25,26]. The problem is how to put these models into practice. One option suggested by some authors is to take a holistic approach, providing clinicians with practical guidance, based on recommendations that may be applied on a case-by-case basis, taking into account personal contexts and preferences, with the participation of patients in decision-making, and focusing on results they consider important [27,28]. An emphasis on congruence between clinician and patient views of treatment goals may be particularly important for the provision of care for complex patients [26].

Despite there being weak evidence on what is the most efficient organisational approach, among possible healthcare initiatives, so-called case management has been proposed as an innovative strategy for improving the quality of life of complex patients, reducing the length of hospital stays, and optimising self-management as well as increasing satisfaction of both patients and professionals [29,30]. However, from the point of view of nurses, the implementation of case management produces many ambiguities and conflicts related to their own role. This was clearly illustrated in our study, with their demand for stability and security, for the role to be definitively established at one or other level of care, and for formal mediation to manage conflicts and resistance in the organisation.

The main factors that determine the results of case management include: a clear definition of the responsibilities of case managers regarding patients they are going to manage, with clarity about their roles and support to ensure that they have the right skills; reliable and valid mechanisms for identifying cases; adequate nurse-to-patient assignment ratios, to ensure that patients and caregivers receive optimal care; shared systems for recording data that can be accessed by the rest of the care team; the widest possible integration of health and social services; and the involvement of stakeholders during the implementation of case management; as well as, evidently, a strong emphasis in the organisation on ensuring continuity of care and self-management education [31]. However, case management alone cannot solve the huge challenges we face. As underlined by Chris Ham [32], it is important to act on several fronts at the same time, effectively promoting case management, but also developing self-management programmes in parallel, and persistently strengthening primary care, together with the essential harmonisation of strategies for change and policies focused on chronicity.

## Conclusion

We have described the perceptions and experiences of clinicians involved in the piloted model.

Analysis of proposals stimulated by the need to find solutions can contribute to improving our understanding of the changes necessary to turn the focus of organisations towards chronic patient care. The results of this study confirm the feasibility of some changes, though they are still limited in extent, and also suggest how further changes could be implemented. In the near future, greater development of elements of the chronic care model in the Goierri-Alto Urola Integrated Healthcare Organisation will require new steps:

1. Primary care nurses will be assigned an advanced case management role and, for this, training has been organised to enable them to develop the necessary skills. Primary care nurses were not involved in the pilot but have participated in later phases especially in the redesign of the project and they have been contributing a point of view not previously considered.
2. To improve care and monitoring of stable patients, given the difficulty of providing protocols to fully cover the needs of patients with multiple chronic conditions (as explained in the Discussion), organisational protocols and patient safety measures will be further developed to ensure that patients receive follow-up that is appropriate to their clinical status.
3. Team work among internists and general practitioners is to be strengthened, using strategies designed with input from members of both groups, and the designation of an assigned internist for each of the seven primary care centres of the OHI. Further, one general practitioner in each primary care centre will be made responsible for the multiple chronic diseases project, and they will coordinate and drive forward the work in joint clinical sessions.
4. Communication with patients will be improved and their safety enhanced by general practitioners preparing a summary report of the clinical assessment of each patient and this being made available to hospital doctors and nurses (internal medicine specialists, and those working in the emergency department and hospital-at-home service).
5. A care plan will be prepared for patients explaining the type of care they will receive, this having been agreed by consensus among all the stakeholders.
6. A process will be set up by which social workers will coordinate with internists to anticipate possible social problems of these patients.
7. The coordination between the hospital-at-home and primary care teams (doctors and nurses) will be intensified, using strategies designed jointly by both parties, with the goal of reducing avoidable admissions of these patients.
8. To lead integration projects and facilitate the participation of all relevant staff, an office of medical services will be created, covering both doctors in the hospital, in internal medicine and other medical specialties, and those in general practice.
